# The Effects of Carbon Source and Growth Temperature on the Fatty Acid Profiles of *Thermobifida fusca*

**DOI:** 10.3389/fmolb.2022.896226

**Published:** 2022-06-01

**Authors:** Dirk C. Winkelman, Basil J. Nikolau

**Affiliations:** Department of Biochemistry, Biophysics and Molecular Biology and the Center of Metabolic Biology, Iowa State University, Ames, IA, United States

**Keywords:** *Thermobifida fusca*, *Actinomycete*, fatty acid biosynthesis pathway, principal component analysis, gas chromatography- mass spectrometry, branched chain fatty acids, fatty acid synthase

## Abstract

The aerobic, thermophilic *Actinobacterium*, *Thermobifida fusca* has been proposed as an organism to be used for the efficient conversion of plant biomass to fatty acid-derived precursors of biofuels or biorenewable chemicals. Despite the potential of *T. fusca* to catabolize plant biomass, there is remarkably little data available concerning the natural ability of this organism to produce fatty acids. Therefore, we determined the fatty acids that *T. fusca* produces when it is grown on different carbon sources (i.e., glucose, cellobiose, cellulose and avicel) and at two different growth temperatures, namely at the optimal growth temperature of 50°C and at a suboptimal temperature of 37°C. These analyses establish that *T. fusca* produces a combination of linear and branched chain fatty acids (BCFAs), including *iso*-, *anteiso*-, and 10-methyl BCFAs that range between 14- and 18-carbons in length. Although different carbon sources and growth temperatures both quantitatively and qualitatively affect the fatty acid profiles produced by *T. fusca*, growth temperature is the greater modifier of these traits. Additionally, genome scanning enabled the identification of many of the fatty acid biosynthetic genes encoded by *T. fusca*.

## Introduction

Plants possess the photosynthetic ability to chemically reduce atmospheric carbon dioxide and generate lignocellulosic biomass, providing the world with a feedstock that could be utilized for production of bio-based chemicals or biofuels (Zoghlami and Paës, 2019). Because plant lignocellulosic biomass can be derived from agricultural waste, it can serve as a feedstock without compromising global food security (Brethauer and Studer, 2014; Li et al., 2021). Fatty acids are a class of energy-dense biomolecules that are similar to petroleum-derived fuels and chemicals, making them potential replacements of fossil-carbon products currently in the marketplace if they can be produced from biorenewable feedstocks (Nikolau et al., 2008; Janssen and Steinbüchel, 2014; Shanks and Keeling, 2017). Unfortunately, this process is hindered by the composition of plant lignocellulosic biomass (i.e., a mixture of cellulose, hemicelluloses, and lignin), which is difficult to catabolize and naturally recalcitrant to microbial and enzymatic degradation (Zoghlami and Paës, 2019). Current methods for breaking down lignocellulosic biomass are costly as they require chemical pretreatments, which inhibit subsequent enzymatic catabolism and add to economic infeasibility (Isikgor and Becer, 2015). Several lignocellulosic degrading microbes are under consideration to serve in consolidated bioprocessing (CBP) strategies in an effort to lower costs (Xiong et al., 2018). A CBP approach would take advantage of a microbe’s natural cellulolytic capabilities and allow simultaneous fermentation of derived sugar monomers to synthesize the desired bioproducts, such as fatty acids.

*Thermobifida fusca* is a thermophilic, cellulolytic *Actinobacterium* that is capable of breaking down lignocellulose. It naturally resides in warmer organic materials, including manure piles, compost heaps, and rotting hay (Mccarthy and Cross, 1984; Zhang et al., 1998). Its ability to hydrolyze plant biomass at higher temperatures (optimum growth at 50°C) and grow over a broad pH range makes it a prime candidate for larger scale CBP applications. The readily available *T. fusca* genome sequence reveals that it has the capacity to express many enzymes useful for hydrolyzing biomass, including numerous cellulases, xylanases, and carbohydrate transporters for sugar uptake (Lykidis et al., 2007). Many of these thermally stable enzymes have been heterologously expressed in alternative microbial hosts and analyzed for their applicability to biomass conversion (Ghangas and Wilson, 1987; Ali et al., 2015; Klinger et al., 2015; Saini et al., 2015; Zhao et al., 2015; Setter-Lamed et al., 2017; Yan and Fong, 2018; Ali et al., 2020).

Although *T. fusca* shows a high propensity to degrade plant biomass, little is known about the fatty acid products that it naturally produces or the fatty acid biosynthetic machinery that the microbe possesses. In this manuscript, we identify many of the fatty acid biosynthetic genes encoded by the *T. fusca* genome. Furthermore, we have determined the fatty acid profiles of *T. fusca* when it is grown on four different carbon sources (i.e., glucose, cellobiose, cellulose, and avicel) at both the optimal growth temperature (50°C) or at a suboptimal temperature (37°C). *T. fusca* has the ability to produce linear saturated and unsaturated fatty acids, but primarily produces a suite of branched-chain fatty acids (BCFAs), particularly *iso*-, *anteiso*-, and 10-methyl BCFAs that are primarily between 14- and 18-carbons in length. In addition, *T. fusca* fatty acid profiles can be affected by environmental changes associated with carbon source and growth temperature, with the latter being the more significant factor driving the fatty acid composition.

## Materials and Methods

### *Thermobifida fusca* Media and Growth Conditions

*T. fusca* (strain BAA-629) was obtained from the American Type Culture Collection (ATCC) (Manassas, Virginia). The frozen stock was revived as directed by ATCC using their standard TYG 741 media, and cultures were incubated at 50°C. Experimental cultures were grown in 100 ml of Hagerdahl media (ATCC medium 2382) supplemented with 0.5% (w/v) of a carbon source: glucose, cellobiose, cellulose, or avicel. Each culture was initiated with 3 ml of inoculum and cultures were grown at either 37°C or 50°C for up to 2 days.

### Harvesting *Thermobifida fusca* Cells

Cells were collected from each culture by centrifugation at 5000 × g for 5 min, and the wet weight of the cell pellet was determined. Cultures grown at the suboptimal temperature (37°C) did not consume all of the insoluble solid carbon source (i.e., cellulose or avicel). These cell pellets were washed with sterile water and the final, washed cell pellet was weighed. Cell pellets were flash frozen in liquid nitrogen, lyophilized for 48 h, and the dry weight was recorded prior to fatty acid extraction.

### Fatty Acid Analysis

Fatty acids were extracted from three aliquots of cells taken from a *T. fusca* culture. Cells were pelleted and lyophilized. Lyophilized cell pellet aliquots (10 mg each) were transferred to a glass tube and spiked with 10 µg of nonadecanoic acid as an internal standard. The pellets were then suspended by vortexing for 15 min in a solution of 5% (v/v) sulfuric acid in methanol and the suspension was incubated at 80°C for 1 h. After cooling to room temperature, 1 ml of hexane: chloroform (4:1 v/v) solution and 1 ml of 0.9% (w/v) NaCl were added to each tube, and the mixture was vortexed for 5 min. The organic and aqueous phases were separated by centrifugation, and the organic phase containing the resulting fatty acid methyl esters was transferred to a separate test tube. The aqueous phase was extracted an additional time with hexane: chloroform (4:1 v/v) solution, and the organic phases were collected and pooled. The extracts containing fatty acid methyl esters were concentrated by evaporation under a stream of nitrogen gas. GC-MS analysis was performed with an Agilent 6890 GC equipped with a DB-1 MS capillary column (Agilent 122–0112). Chromatography was performed using helium gas at a flow-rate of 1.2 ml/min, using an inlet temperature set at 280°C. Individual fatty acid methyl esters were identified by GC-MS fragmentation patterns in tandem with NIST AMDIS software (Stein, 1999), as well as by comparing their retention times to known fatty acid methyl ester standards obtained from Supelco Inc. (Bellefonte, PA) and Metraya LLC (State College, PA). Peak areas of individual fatty acid methyl esters were integrated with AMDIS software, and these were converted to fatty acid concentrations relative to the peak area of the known amount of nonadecanoic acid internal standard that was added to each sample.

### Principal Component Analysis

Principal component analysis was conducted using Metaboanalyst software (Pang et al., 2021) with data from all replicates of *T. fusca* cultures grown in each of the eight growing conditions. Quantitative fatty acid abundance data (µmoles/g dry cell weight) were uploaded from Microsoft Excel to Metaboanalyst statistical software and autoscaling was done as enabled by Metaboanalyst to create a PCA plot and a PCA biplot.

### Computational Identification of Enzymatic Components of the *Thermobifida fusca* Fatty Acid Biosynthesis Machinery

The sequenced *T. fusca* genome was queried with the BLASTP algorithm (Altschul et al., 1990) using query sequences of experimentally confirmed acetyl-CoA carboxylase (ACCase) and Type II fatty acid synthase (FAS) components, either originating from *Actinomycetes* or from *Escherichia coli* (Cronan and Thomas, 2009; Shivaiah et al., 2021). Additionally, genes were identified using the KEGG genome browser (Kanehisa et al., 2002) based on sequence homology, key processes, and operon organization.

## Results

### Fatty Acids Produced by *Thermobifida fusca* at Optimal Growing Conditions

**FIGURE 1:**
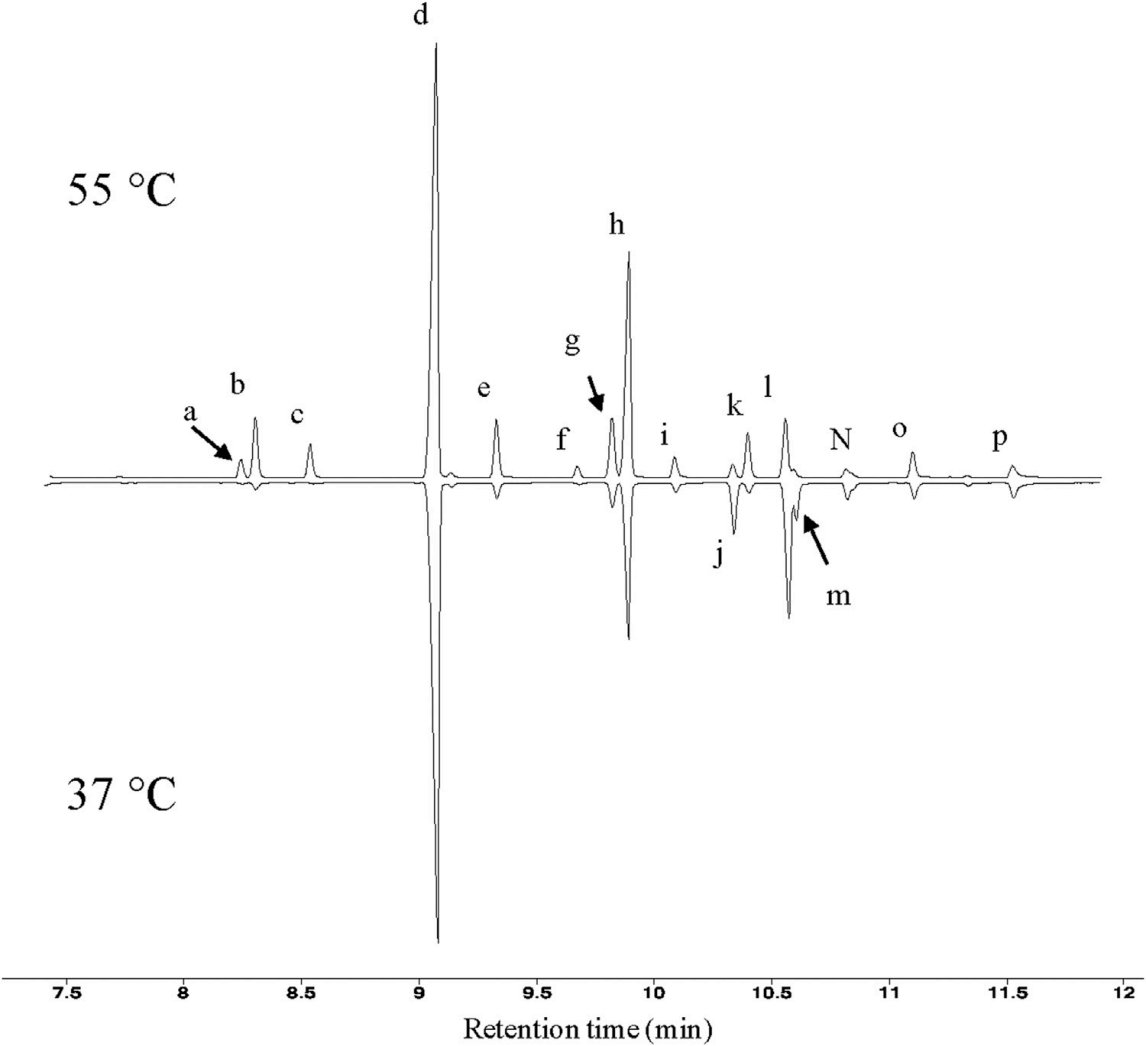
GC profiles of *T. fusca* fatty acids. Typical GC profiles of fatty acid methyl esters isolated from *T. fusca* cultures grown on cellobiose at the indicated growth temperatures. Fatty acids were identified by mass-spectrometry and by comparing retention time with commercial standards. a = *iso*-15:0; b = *anteiso*-15:0; c = *n*-15:0; d = *iso*-16:0; e = *n*-16:0; f = 10-methyl-16:0; g = *iso*-17:0; h = *anteiso*-17:0; i = *n*-17:0; j = unknown; k = 10-methyl-17:0; l = *iso*-18:0; m = unknown; n = *n*-18:0; o = 10-methyl-18:0; *p* = *n*-19:0.

Fatty acid analyses show that when *T. fusca* was grown at 50°C it primarily synthesizes fatty acids that are between 14 and 18 carbons in length, although trace amounts of 13-carbon fatty acids were also detected. The majority of the fatty acids produced were BCFAs, particularly *iso*- or *anteiso*-BCFAs (i.e., the methyl branch is present at the ω-1 or ω-2 position of the acyl chain, respectively) (Figure 1). Small amounts of mid-chain BCFAs were also detected, these being 10-methyl BCFAs ranging between 16 and 18 carbons in length. Linear fatty acids were also present, accounting for approximately 10% of total fatty acids produced. Additionally, we determined that *T. fusca* can produce minor amounts (<5%) of mono-unsaturated C16 and C18 fatty acids with a single double bond at the 9th position.

### Fatty Acid Profiles Are Affected by Carbon Source and Growth Temperature

**FIGURE 2:**
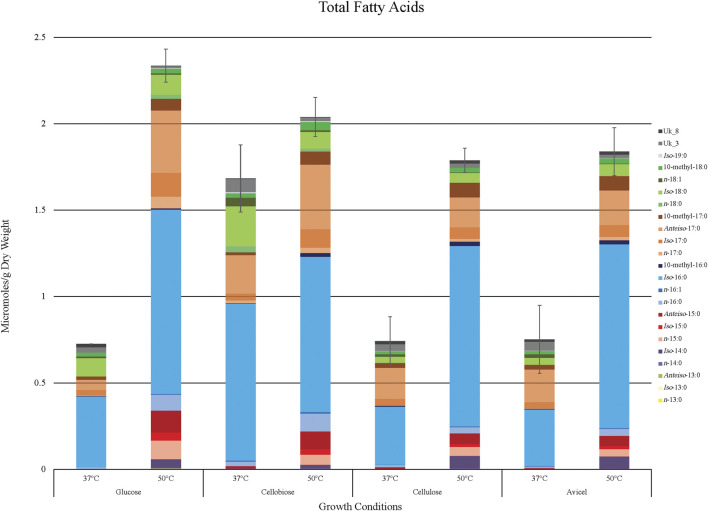
Fatty acid yield generated by *T. fusca* in various growing conditions. Total accumulation of all identified fatty acid products (µmoles/g dry weight) when *T. fusca* was grown on glucose, cellobiose, cellulose, or Avicel as carbon source and cultured at either 37°C or 50°C, respectively. Fatty acid species are stacked in order of increasing chain length. Error bars represent standard error from three replicates.

**FIGURE 3:**
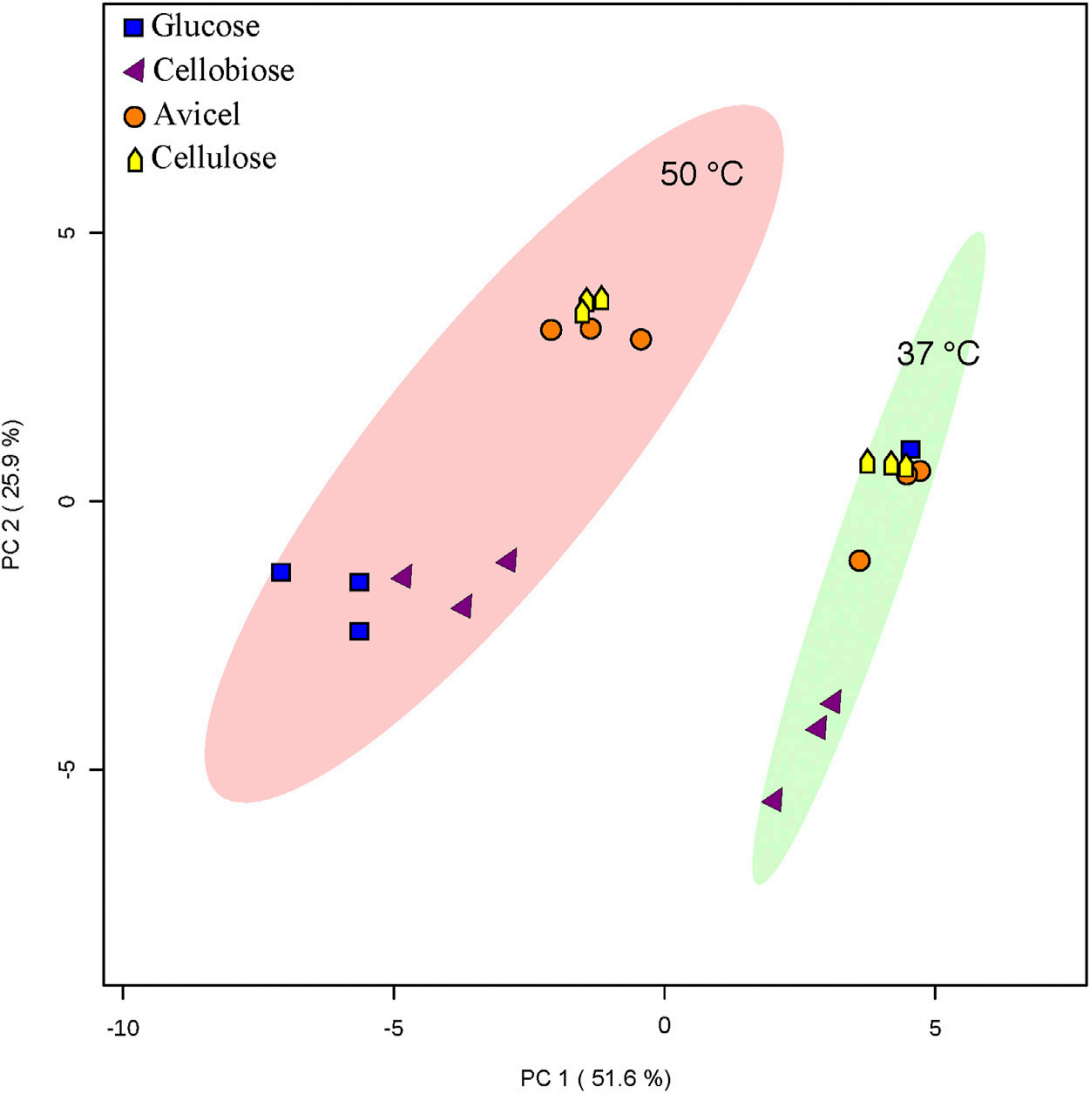
PCA analysis. PCA analysis was conducted with Metaboanalyst software. An ellipse indicating 95% confidence regions for each heterotic group (37°C or 50°C growth temperature) is provided. Only one replicate of *T. fusca* supplemented with glucose at 37°C is depicted.

Overall fatty acid yield was 2- to 3- fold higher when *T. fusca* was grown at the optimal growth temperature (50°C) as compared to growth at the suboptimal temperature (37°C) (Figure 2), and this phenomenon was observed independent of the carbon sources that were evaluated. Principal component analysis of the fatty acid compositional data visualized the factors contributing to the different fatty acid profiles of *T. fusca* through generation of a PCA plot (Figure 3). Principal component 1 accounts for ∼52% of the sample variation, indicating that the samples are primarily separated by growth temperature, whereas carbon source contributed to ∼26% of the sample variation (represented by principal component 2). Indeed, the data points in the PCA plot cluster distinctly by growth temperature, and to a lesser extent by carbon source, with soluble and insoluble carbon sources typically clustering together within each growth temperature cluster. The conclusion that growth temperature is the primary driver of fatty acid composition is indicated by the PCA biplot (Supplementary Figure S1), as most of the vectors representing the different fatty acids are pointed horizontally, indicating that they contributed more to principal component 1. Additionally, some of the longer-chain fatty acids (18-carbons and longer) and the 10-methyl BCFAs are more associated with changes in the carbon source, as their vectors point more vertically.

The fatty acid profile of *T. fusca* shifted when it was cultured at its suboptimal temperature; the relative abundance of BCFAs was increased when the bacterium was cultured at 37°C (Supplementary Figure S2). Growth temperature also affected the acyl-chain lengths of the fatty acids produced by *T. fusca*. While C13, C14, and C15 fatty acid species account for approximately 10–15% of the fatty acids present at 50°C, they are almost completely absent from the cultures grown at 37°C (Supplementary Figure S3). Although unsaturated fatty acids were only detected at trace amounts at the optimum growth temperature, they make up a more significant proportion of the total fatty acid content when *T. fusca* was cultured at 37°C (∼5%).

While growth temperature was the primary factor determining the types of fatty acids produced, carbon source also contributed to a shift in fatty acid profiles. Changes were primarily observed when comparing cultures grown on soluble versus insoluble carbon sources. Specifically, the fatty acid profiles of *T. fusca* grown on cellulose were very similar to those obtained when *T. fusca* was grown on Avicel, while fatty acid profiles of cultures grown on cellobiose resembled those grown on glucose. The main shift between the cultures grown on the soluble versus insoluble carbon sources can be attributed to the types of BCFAs present. While *iso*-BCFAs are the main species present in all growth conditions, when *T. fusca* was grown on soluble carbon sources we observed an even higher proportion of *iso*-BCFAs at 37°C. This relationship is reversed when using insoluble carbon sources; namely there is a higher proportion of *iso*-BCFAs at 50°C. In both cases, the change in abundance of *iso*-BCFAs is complemented with a corresponding change in the abundance of *anteiso*-BCFAs.

### Identification of *Thermobifida fusca* Fatty Acid Biosynthesis Machinery

**FIGURE 4:**
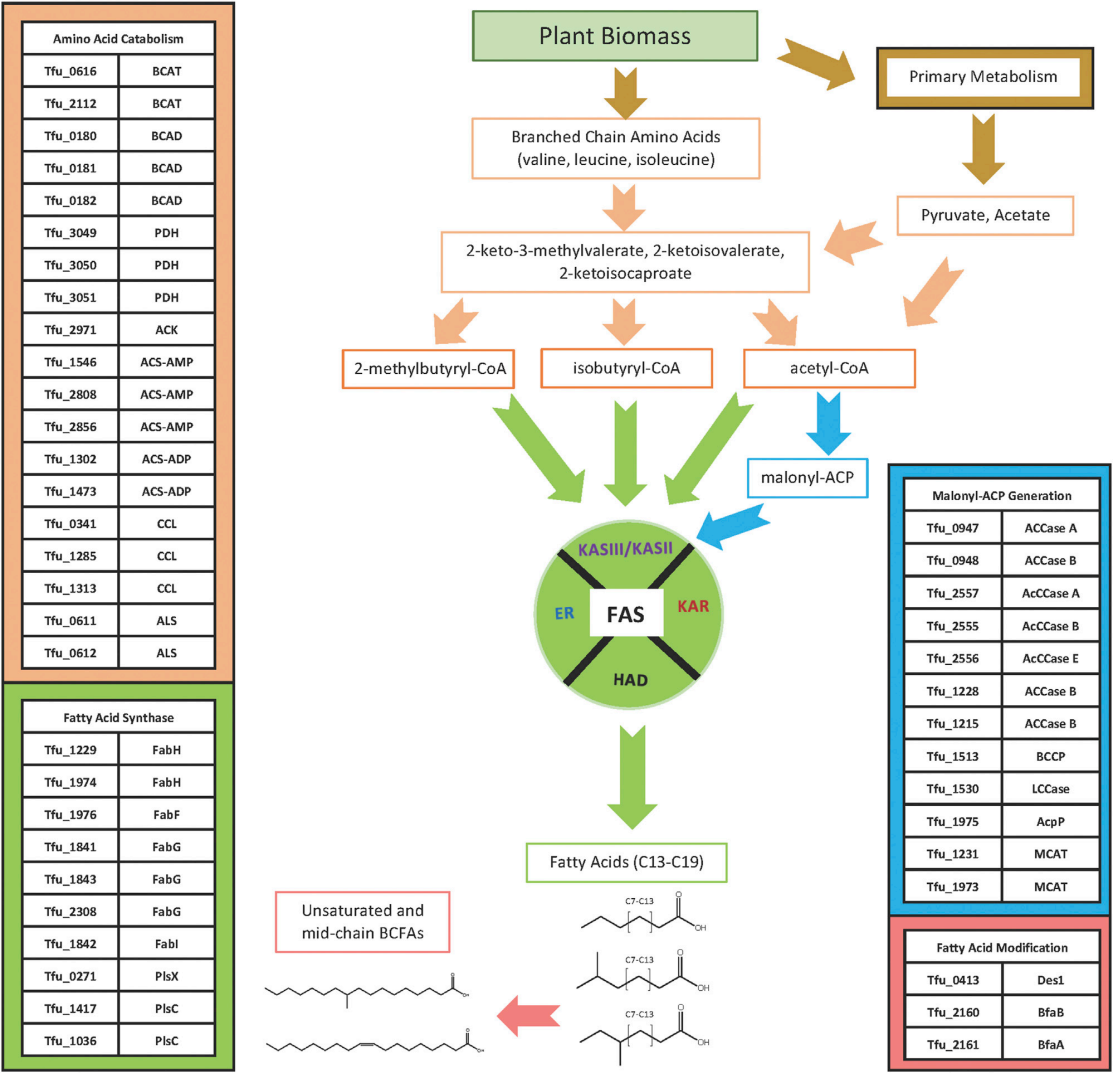
Fatty acid biosynthesis pathway. Acyl-CoA starting substrates for FAS are generated from glycolytic catabolism of glucose or from α-keto acids that can be produced *via* the catabolism of branched-chain amino acids or as the penultimate intermediates in the biosynthesis of branched chain amino acids. The malonyl-ACP substrate used for the elongation reaction of FAS is synthesized from acetyl-CoA by ACCases. The acyl-CoA and malonyl-ACP substrates are used by the FAS system to elongate *iso-*, *ante-iso*, and linear *n-*fatty acids, which can be modified to produce unsaturated and 10-methyl branched chain fatty acids.

Although many of the enzymes involved in fatty acid biosynthesis have not been specifically characterized from *T. fusca*, they are identifiable by their sequence homology to enzymes from other bacteria, and by the operon organization in the *T. fusca* genome (Table 1). Querying the sequence of the *T. fusca* genome indicates that like many other bacteria, *T. fusca* utilizes a Type II fatty acid synthase (FAS) system to assemble fatty acids (Cronan and Thomas, 2009; Gago et al., 2011; Gago et al., 2018). In most organisms, FAS utilizes acetyl-CoA and malonyl-CoA as substrates, and the latter substrate is generated by the carboxylation of the former, a reaction catalyzed by acetyl-CoA carboxylase (Waldrop et al., 2012). Multiple iterations of the FAS cycle using these two substrates generates linear, saturated fatty acids, but these are minor components in *T. fusca*. The *iso*-and *anteiso*-BCFAs that account for a large portion of the fatty acids of *T. fusca* are produced by this FAS system by using branched-chain acyl-CoA substrates, rather than acetyl-CoA, which can be generated by the deamination of branched chain amino acids (i.e., valine, leucine, or isoleucine) (Kaneda, 1991; Beck et al., 2004; Zhu et al., 2005). Thus, the fatty acid biosynthetic machinery of *T. fusca* can be considered as consisting of at least four modules (Figure 4): 1) the module that generates the acyl-CoA starting substrate for FAS; 2) the module that generates the malonyl-CoA elongating substrate for FAS; 3) the FAS system itself; and 4) a fatty acid modifying module which generates the unsaturated and the internally BCFAs.

**TABLE 1:** Identification of *T. fusca* fatty acid biosynthesis machinery. Genes were identified using the BLASTP algorithm using query sequences of experimentally confirmed enzymes from *E. coli* or from *Actinomycetes*.

Enzyme	Description	Gene name
ACCase A	ACCase BC and BCCP subunits	Tfu_0947
ACCase B	ACCase CT subunit	Tfu_0948
AcCCase A	AcCCase BC and BCCP subunit	Tfu_2557
AcCCase B	AcCCase CT subunit	Tfu_2555
AcCCase E	AcCCase E subunit	Tfu_2556
ACCase B	Additional ACCase CT subunit	Tfu_1228, Tfu_1215
BCCP	Biotin Carboxyl-Carrier Protein	Tfu_1513
LCCase	LCCase	Tfu_1530
AcpP	Acyl-carrier protein (ACP)	Tfu_1975
MCAT	Malonyl-CoA:ACP transacylase	Tfu_1231, Tfu_1973
FabH	3-ketoacyl-ACP synthase III	Tfu_1229, Tfu_1974
FabF	3-ketoacyl-ACP synthase III isozyme	Tfu_1976
FabG	3-ketoacyl-ACP reductase	Tfu_1841, Tfu_1843, Tfu_2308
FabA	3-hydroxyacyl-ACP dehydratase	Unknown
FabI	Enoyl-ACP reductase	Tfu_1842
PlsX	Glycerol-3-phosphate acyltransferase	Tfu_0271
PlsC	Glycerol-3-phosphate acyltransferase	Tfu_1417, Tfu_1036
BCAT	Branched chain amino acid aminotransferase	Tfu_0616, Tfu_2112
BCAD	Branched Chain alpha keto acid dehydrogenase	Tfu_0180, Tfu_0181, Tfu_0182
Des1	Delta-9 acyl-CoA desaturase	Tfu_0413
BfaB	Δ9 unsaturated fatty acid methyl transferase	Tfu_2160
BfaA	10-methylene BCFA reductase	Tfu_2161
PDH	Pyruvate Dehydrogenase Complex	Tfu_3049, Tfu_3050, Tfu_3051
ACK	Acetate Kinase	Tfu_2971
ACS-AMP	AMP-Forming Acetyl-CoA Synthetase	Tfu_1546, Tfu_2808, Tfu_2856
ACS-ADP	ADP-Forming Acetyl-CoA Synthetase	Tfu_1302, Tfu_1473
CCL	Citryl-CoA lyase	Tfu_0341, Tfu_1285, Tfu_1313
ALS	Acetolactate synthase	Tfu_0611, Tfu_0612

The acyl-CoA substrates required by this organism’s FAS system are products of primary metabolism from sugars, generating acetyl-CoA, as well as products of branched chain amino acid metabolism, generating isobutyryl-CoA and 2-methylbutrylyl-CoA. Sequence homology identified multiple candidates for both the branched chain aminotransferase (Tfu_0616, Tfu_2112) and branched chain α-keto acid dehydrogenase (Tfu_0180, Tfu_0181, Tfu_0182) enzymes required to generate isobutyryl-CoA and 2-methylbutyryl-CoA from valine, leucine, or isoleucine. Alternatively, these branched chain acyl-CoAs may be generated from the α-keto acids that are intermediates of branched chain amino acid biosynthesis. Indeed, the branched chain aminotransferase encoded by Tfu_0616 is located in the genome adjoining genes that encode enzymes involved in branched chain amino acid biosynthesis, including genes with high sequence homology to acetolactate synthase (Tfu_0611, Tfu_0612), keto-acid isomeroreductase (Tfu_0613), 3-isopropylmalate dehydrogenase (Tfu_0615), 2-isopropylmalate synthase (Tfu_0617), and 3-isopropylmalate dehydratase (Tfu_0626, Tfu_0627). (Franco and Blanchard, 2017). Acetyl-CoA can be produced through several biological processes (Krivoruchko et al., 2015), including the oxidative decarboxylation of pyruvate catalyzed by the pyruvate dehydrogenase complex (PDH) (Tfu_3049, Tfu_3050, Tfu_3051). Alternatively, acetyl-CoA can be generated through the activation of acetate catalyzed by: 1) an acetate kinase (Tfu_2971); 2) an AMP-forming acetyl-CoA synthetase (Tfu_1546, Tfu_2808, Tfu_2856); or 3) an ADP-forming acetyl-CoA synthetase (Tfu_1302, Tfu_1473). *T. fusca* also possesses three genes that encode for proteins that resemble citryl-CoA lyase (Tfu_0341, Tfu_1285, Tfu_1313), a component of the reductive TCA cycle capable of converting citryl-CoA to acetyl-CoA and oxaloacetate (Aoshima et al., 2004; Hügler et al., 2005; Hügler et al., 2007; Katiyar et al., 2018).

The biotin-containing enzyme, acetyl-CoA carboxylase (ACCase) converts acetyl-CoA to malonyl-CoA, a reaction that is classically considered the first and rate-limiting reaction of fatty acid biosynthesis. As with all biotin enzymes, sequences of these proteins can be recognized by sequence homology among three different functional domains: the biotin carboxylase (BC), biotin carboxyl carrier protein (BCCP), and the carboxyl transferase (CT) domains (Cronan and Waldrop, 2002; Tong, 2013). The tertiary and quaternary organization of these domains varies considerably, depending on the phylogeny of the organism. *E. coli*, for examples, has ACCase components that are organized as individual proteins that come together to form the enzyme complex. In contrast, several ACCases from *Actinomycetes* consist of two subunits: the A subunit that encompasses both the BC and BCCP functional domains, and the B subunit that encompasses the CT domain (Gago et al., 2011; Gago et al., 2018). Additionally, some *Actinomycete* ACCases have a third non-catalytic subunit, E, that is needed for proper assembly of the holoenzyme complex (Shivaiah et al., 2021). Moreover, the A and B subunit quaternary organization of biotin enzymes is also common to propionyl-CoA carboxylase (Tong, 2013) and methylcrotonyl-CoA carboxylase (Song et al., 1994; McKean et al., 2000; Wurtele and Nikolau, 2000), which complicates the sequence-based identification of ACCase in the *T. fusca* genome.

Previous studies have experimentally characterized the *T. fusca* operon (Tfu_2555, Tfu_2556, Tfu_2557) that encodes the B, E, and A subunits of an acyl-CoA carboxylase (AcCCase) (Shivaiah et al., 2021). This enzyme is promiscuous and can carboxylate acetyl-CoA, propionyl-CoA, and butyryl-CoA. Other *Actinobacteria* (e.g., *Streptomyces coelicolor*) also express such a promiscuous carboxylase, in addition to a highly specific propionyl-CoA carboxylase (Gago et al., 2018). The more promiscuous AcCCase enzymes can thereby generate not only malonyl-CoA, but also methylmalonyl-CoA and ethylmalonyl-CoA, and may therefore have multiple metabolic functions. For example, the catabolism of valine, isoleucine and odd-numbered fatty acids generates propionyl-CoA, which is further metabolized *via* the TCA cycle after the sequential conversion to methylmalonyl-CoA and succinyl-CoA (Wongkittichote et al., 2017). Alternatively, methylmalonyl-CoA and ethylmalonyl-CoA can be used as substrates by polyketide synthases, generating polyketides with methyl- or ethyl-branches in the final structure (Khosla and Keasling, 2003; Risdian et al., 2019).

Our BLAST-based search of the *T. fusca* genome identified additional genes that encode homologs of biotin-containing carboxylase proteins, namely Tfu_0947, Tfu_0948, Tfu_1215, Tfu_1228, Tfu_1513, and Tfu_1530. The adjacent Tfu_0947 and Tfu_0948 genes suggest that they are on a single operon, with Tfu_0947 encoding a subunit with the BC and BCCP domains, and Tfu_0948 encoding a subunit carrying the CT domain. This subunit/domain organization suggests that this operon may encode either a propionyl-CoA carboxylase (Gago et al., 2018) or methylcrotonyl-CoA carboxylase (Tomassetti et al., 2018), although ACCases with such quaternary subunit organizations also occur in many *Actinomycetes*, including *Streptomyces coelicolor* and *Mycobacterium tuberculosis* (Gago et al., 2011; Tong, 2013).

The Tfu_1228 and Tfu_1215 genes encode proteins that resemble CT subunits of biotin-carboxylases, but neither gene lies within an operon that houses other functional subunits necessary to form the holoenzyme complex. It is a common feature among *Actinomycetes* to mix and match different CT-subunits with a common BC/BCCP subunit, and thus generate different enzymatic capability with a single BC/BCCP subunit (Gago et al., 2011; Gago et al., 2018). The Tfu_1228 and Tfu_1215 genes may instill such a mechanism, and thus these genes could also provide a means for generating malonyl-CoA for FAS. A similar mechanism may occur in *T. fusca*, as Tfu_1228 and Tfu_1215 could be part of additional ACCase complexes that use an A subunit from another operon (such as Tfu_2557 or Tfu_0947). An additional gene with sequence homology to known ACCase components is Tfu_1513, which encodes a protein with high sequence homology to a BCCP that is not located near a BC or CT domain. Such a genome organization is similar to that found in *E. coli*, where the BC, BCCP, and CT components are separated into four individual proteins encoded by 3 separate operons (Cronan and Waldrop, 2002; Gago et al., 2018).

The Tfu_1530 gene encodes a large protein of 1849 residues, and it appears to encompass all three catalytic domains required for the carboxylation reaction (i.e., BC, BCCP and CT domains). This homomeric domain organization is common to such biotin carboxylases as ACCases from eukaryotes (i.e., plants, fungi, and animals) (Nikolau et al., 2003; Sasaki and Nagano, 2004), pyruvate carboxylases (Jitrapakdee and Wallace, 1999) and a long chain acyl-CoA carboxylase from *Mycobacterium* species (Tran et al., 2015; Lyonnet et al., 2017). The latter enzyme is involved in the biosynthesis of mycolic acid, a fatty acid specifically associated with the *Mycobacterium* genus (Marrakchi et al., 2014). Thus, the specific enzymatic function encoded by the Tfu_1530 gene is not recognizable by sequence homology but may include the ability to generate malonyl-CoA for FAS.

Type II FAS systems use cyclic iterations of four reactions that are each catalyzed by distinct enzymes (Cronan and Rock, 2008). Each cycle of the process adds two carbon atoms to the growing acyl-chain, with the donor of these two carbon subunits being the malonyl moiety of malonyl-CoA. The malonyl-moiety is first loaded onto the acyl-carrier protein (ACP) subunit of the FAS system, a reaction catalyzed by malonyl-CoA: ACP transacylase (MCAT). Subsequently, each FAS cycle begins with a Claisen condensation reaction between a preexisting “starting” acyl-CoA or acyl-ACP and malonyl-ACP to generate a 3-ketoacyl-ACP intermediate, which is 2-carbons longer than the initial acyl moiety. The first of these Claisen condensation reactions is between an acyl-CoA and malonyl-ACP, catalyzed by a 3-ketoacyl-ACP synthase III (encoded by the *FabH* gene). The chemical nature of the acyl-CoA substrate used in this condensation reaction determines the nature of the ω-end of the resulting fatty acid product; namely, utilizing acetyl-CoA, isobutyryl-CoA or methylbutyryl-CoA as the substrate leads to the generation of linear, *iso*-BCFA or *anteiso*-BCFA, respectively. The subsequent three reactions of each FAS cycle involve sequential reduction, dehydration and further reduction, catalyzed by 3-ketoacyl-ACP reductase (*FabG*), 3-hydroxyacyl-ACP dehydratase (*FabA*), and enoyl-ACP reductase (*FabI*), respectively. The product of each FAS cycle results in the generation of an acyl-ACP product that is two carbons longer than the pre-loaded acyl chain, and it serves as the substrate for the Claisen condensation reaction of the next round of the FAS cycle; these subsequent Claisen condensation reactions with a malonyl-ACP substrate are catalyzed by a 3-ketoacyl-ACP synthase II isozyme (*FabF*). These catalytic processes generate saturated fatty acids, and typically the process is terminated by transfer of the acyl moiety from acyl-ACP to glycerol-3-phosphate (glycerol-3-P), a reaction catalyzed by glycerol-3-P acyltransferases (GPATs) (Yao and Rock, 2013). The substrate specificity of GPATs determine the chain-lengths of the fatty acids produced by FAS, and in most bacteria they are typically of between 14 and 18 carbon atoms.

The *T. fusca* genome contains multiple operons that could encode for FAS genes, although the similarity to enzymatic functions associated with polyketide biosynthesis, catalyzed by Type II polyketide synthase or a nonribosomal peptide biosynthetic system (Corre and Challis, 2009), may confound these identifications. Specifically, Tfu_1973 (MCAT) is part of a large operon that includes Tfu_1974 (*FabH*), Tfu_1975 (*ACP*), and Tfu_1976 (*FabF*). Another copy of MCAT is encoded by Tfu_1231, which is also positioned near an additional copy of *FabH* (Tfu_1229). We found three *FabG* genes in the *T. fusca* genome that appear to encode for the 3-ketoacyl-ACP reductase. Two of these *FabG* genes, Tfu_1841 and Tfu_1843, are located in the same operon that also contains Tfu_1842 (*FabI*). A third copy of *FabG* (Tfu_2308), is found separately and does not appear to be part of a larger operon.

The fatty acid modification module includes genes that are required to generate unsaturated and 10-methyl BCFAs. Recent studies have indicated that *Actinomycetes* express a three-reaction pathway that metabolically links these two fatty acids. Specifically, a Δ-9 acyl-CoA desaturase (Tfu_0413) (Lykidis et al., 2007) can generate monounsaturated fatty acids, which are substrates for BfaB (Tfu_2160) and BfaA (Tfu_2161) enzymes, which transform the monounsaturated acyl-CoA to 10-methylene BCFA and to 10-methyl BCFA, respectively (Blitzblau et al., 2021).

## Discussion

While plants have presented the world with a large renewable feedstock of lignocellulosic biomass, the recalcitrant nature of this material has provided a challenge to make its utilization economically feasible (Isikgor and Becer, 2015; Zoghlami and Paës, 2019; Li et al., 2021). Fortunately, biological evolution has produced organisms capable of breaking down plant biomass (Brethauer and Studer, 2014; Saini et al., 2015; Xiong et al., 2018). One such organism that has received attention for its ability to catabolize biomass is *T. fusca* (Lykidis et al., 2007; del Pulgar and Saadeddin, 2014; Deng et al., 2016; Vanee et al., 2017). However, the breakdown of lignocellulosic biomass must be coupled to the conversion of the derived carbon to molecular structures that have utility as replacements of fossil-carbon based chemicals, fuels and materials. Biologically produced fatty acids have chemo-physical properties that make them highly desirable as substitutes of fossil-carbon products (Nikolau et al., 2008; Janssen and Steinbüchel, 2014; Shanks and Keeling, 2017). We have therefore evaluated the types of energy-dense fatty acid molecules that *T. fusca* is capable of producing.

Specifically, we have profiled the fatty acids that *T. fusca* produces in eight different growth conditions, and in parallel we have queried the sequenced genome of this organism to identify many of the genes that may be involved in the conversion of lignocellulosic-derived carbon to fatty acids. In these analyses fatty acids were chemically converted to methyl esters, which facilitated their subsequent GC-based identification and quantification. Because this conversion was based on transmethylation chemistry, which converts existing fatty acyl esters to methyl esters, we infer that the fatty acids that we profiled are acyl moieties of more complex lipids. Such lipids could include membrane associated polar lipids (e.g., phospholipids) or non-polar storage lipids (e.g., triacylglycerols). Although some *Actinomycetes* are capable of producing triacylglycerols, the absence of a gene for diacylglycerol acyltransferases in the *T. fusca* genome (Lykidis et al., 2007) that is necessary for triacylglycerol biosynthesis would preclude the occurrence of these lipids. Moreover, in *Actinomycetes,* triacylglycerols usually accumulate during the stationary phase of growth (Olukoshi and Packter, 1994; Alvarez and Steinbüchel, 2002; Comba et al., 2013; Santucci et al., 2019). Thus, we surmise that the fatty acids identified in this study are primarily membrane-associated phospholipids, which accumulate to facilitate the exponential growth of bacteria.

The primary fatty acids of *T. fusca* are three types of BCFAs: *iso*-branched, *anteiso*-branched, and mid-chain branched. We evaluated the metabolic responsiveness of *T. fusca* in relation to different growth temperature and different carbon sources and found that the fatty acid profiles shifted particularly in response to growth temperature. This temperature induced change in the fatty acid profile is consistent with the need to maintain membrane fluidity at the cooler temperature and results in increased proportion of BCFAs (Mansilla et al., 2004; Mendoza, 2014), although this shift in fatty acid composition is also dependent on carbon source. For example, when grown on glucose or cellobiose, there is a proportional increase in *iso*-BCFAs at 37°C. In many organisms, maintaining membrane fluidity at lower temperatures is achieved by increasing the degree of fatty acid unsaturation, which also occurs in *T. fusca*. Because the proportion of unsaturated fatty acids was always <5% of the total recovered fatty acids, it is difficult to envision that the unsaturated fatty acids are solely responsible for maintaining membrane fluidity at the cooler temperature. Thus, as with other microbial species (Klein et al., 1999; Saunders et al., 2016; Hassan et al., 2020), we suggest that the alteration in BCFAs is the main mechanism by which *T. fusca* maintains membrane fluidity at colder temperatures.

BFCAs have chemo-physical attributes that make them of particular interest for engineering applications. Specifically, the methyl-branch in the alkyl chain has the effect of lowering the melting point of the fatty acid, as compared to the linear-chain fatty acids of the same number of carbon atoms (Yao and Hammond, 2006). Therefore, BCFAs can maintain a fluid state at lower temperatures, which is desirable for biodiesel or lubrication application purposes in colder climates (Bart et al., 2013). Although unsaturated fatty acids can be used for these purposes, they are more susceptible to oxidation in these applications that expose them to oxygen at higher temperatures and pressures, as occurs in combustion engines. Thus, *T. fusca* can be viewed as a source of saturated BCFAs, with enhanced application potential as biofuels and biolubricants.

Despite this potential for producing desirable bioproducts, the yield of fatty acids from *T. fusca* cultures is relatively low as compared to such oleaginous organisms, such as plant oil seed crops (e.g., sunflower, canola, safflower), yeasts (e.g., *Yarrowia lipolytica* and *Rhodosporidium toruloides*) and microalgae (e.g., *Botryococcus braunii*). These later organisms hyperaccumulate fatty acid containing neutral lipids that can account for 20–60% of the dry biomass (Banerjee et al., 2002; Sharafi et al., 2015; Patel et al., 2019; Liu H. et al., 2021; Liu Y. et al., 2021; Petraru et al., 2021; Zemour et al., 2021). In contrast, *T. fusca* accumulates fatty acids at approximately 1 mg/g dry biomass. Increasing fatty acid titers from non-model organisms such as *T. fusca* is becoming increasingly viable, either by mutagenesis and selection strategies (Guo et al., 2019; Südfeld et al., 2021), or direct targeted genetic engineering strategies (Zhang et al., 2019; Racharaks et al., 2021). The latter strategy needs prior knowledge of the fatty acid biosynthesis machinery that would be targeted for genetic engineering, whereas the former strategy can be informative of regulatory mechanisms, once mutant alleles can be genetically mapped relative to the fatty acid biosynthesis machinery encoded by the target genome. We therefore examined the *T. fusca* genome to identify the components of the fatty acid biosynthesis machinery, which would facilitate these strategies for improving fatty acid yields.

*T. fusca* appears to possess much of the fatty acid biosynthesis machinery that is common to other bacteria, particularly *Actinomycetes* (Gago et al., 2011; Lyonnet et al., 2017; Gago et al., 2018). At the core is the Type II FAS system, which appears to be encoded primarily within two operons, encompassing the genes Tfu_1973 to Tfu_1975 and Tfu_1841 to Tfu_1843. Additional homologs of some of the FAS enzymatic components are also encoded in the genome, but these may be associated with a polyketide synthase system or the nonribosomal peptide biosynthetic system responsible for the biosynthesis of the siderophore, fuscachelins (Dimise et al., 2008; Corre and Challis, 2009).

The FAS system needs to be provided with four different substrates: acetyl-CoA, isobutyryl-CoA, 2-methylbutyryl-CoA and malonyl-CoA. The former substrates are used to initiate FAS reactions, and thereby generate linear fatty acids and two types of BCFAs; whereas malonyl-CoA is the substrate that is used to elongate the fatty acid in the FAS catalyzed reaction. We identified five potential enzymatic systems that can generate acetyl-CoA: the pyruvate dehydrogenase complex, AMP-forming acetyl-CoA synthetase, ADP-forming acetyl-CoA synthetase, acetate kinase, and citryl-CoA lyase. These systems give *T. fusca* the ability to produce acetyl-CoA from multiple carbon sources, including pyruvate and acetate. Citryl-CoA lyase represents the final step in the reductive TCA cycle that also requires a 2-oxoglutarate: ferredoxin oxidoreductase (Tfu_2674, Tfu_2675). The presence of these enzymes potentially gives *T. fusca* the ability to synthesize acetyl-CoA from carbon dioxide (Aoshima et al., 2004; Hügler et al., 2005), a process that has previously been explored in *Mycobacterium tuberculosis* (Boshoff and Barry, 2005; Watanabe et al., 2011; Katiyar et al., 2018). The reductive TCA cycle can be considered the reversal of the oxidative TCA cycle that is prevalent in aerobic organisms and oxidizes carbon from acetyl-CoA to generate carbon dioxide. The reductive TCA cycle is considered to be a primordial pathway for carbon dioxide fixation facilitating autotrophic metabolism in the earliest life-forms on earth, possibly prior to the advent of the currently prevalent photosynthetic pentose phosphate cycle (Buchanan and Arnon, 1990; Romano and Conway, 1996; Smith and Morowitz, 2004). Thus, *T. fusca* has the metabolic flexibility for generating this important intermediate in metabolism, and moreover these mechanisms provide opportunities for improving acetyl-CoA generation *via* genetic engineering strategies, as has been done in a number of microbial systems (Lian et al., 2014; Liu et al., 2017; Soma et al., 2017; Ku et al., 2020).

Several biotin-containing enzyme systems were identifiable *via* sequence homology, which could generate the malonyl-CoA substrate that is used by FAS to elongate the fatty acid. These enzymes carboxylate acyl-CoA substrates, including acetyl-CoA, and thereby generate malonyl-CoA (Nikolau et al., 2003), which is considered to be the rate-limiting reaction for fatty acid biosynthesis (Davis et al., 2000; Chaturvedi et al., 2021). Prior experimental characterization has identified one of these biotin-containing enzymes as being capable of carboxylating acetyl-CoA, propionyl-CoA, or butyryl-CoA (Shivaiah et al., 2021), but whether *T. fusca* encodes other proteins that can also carboxylate acetyl-CoA will require similar characterizations.

Finally, the *T. fusca* genome features enzymes capable of modifying fatty acids following their assembly by the FAS system. These include an acyl-CoA desaturase to produce unsaturated fatty acids, which are substrates for a methylase and reductase needed to generate 10-methyl BCFAs (Blitzblau et al., 2021). These three enzymes, in addition to the flexibility of the FAS system to use an assortment of α-carboxyl acyl-CoAs, indicate that *T. fusca* possesses the metabolic machinery to convert plant biomass to a wide array of fatty acids that have applications as biorenewable products. In particular, *T. fusc*a appears to be an organism that can generate unique combinations of BCFAs, including those with a mid-chain branch, which have potential applications as feedstocks for novel bioproducts, such as bio-lubricants (Blitzblau et al., 2021).

Collectively the fatty acid profiling data integrated with the genomics data identified the genetic potential of *T. fusca*. Additional data that evaluates the expression of this genetic potential would precisely deduce the catalytic and regulatory circuit(s) that define the metabolic system that generates the diversity of fatty acids produced by this organism. Such genetic expression data will require extensive steady state transcriptomics, proteomics and metabolomics data, integrated with flux analyses to further expand the comprehension of the metabolic system that converts sugar-feedstocks to fatty acids, and thereby make *T. fusca* an even more attractive candidate to produce energy-dense biomolecules in a consolidated bioprocessing system.

## Data Availability

The original contributions presented in the study are included in the article/Supplementary Material, further inquiries can be directed to the corresponding author.

## Supplementary Material

Supplementary Figure S1 PCA biplot of T. fusca fatty acid data. Overlay of PCA score plot and loading plot. Cultures numbered 1-3 were grown at 37°C, while cultures numbered 4-6 were grown at 50°C. CB, cellobiose, GL, glucose, CL, cellulose, AV, avicel. Only a single determination of *T. fusca* supplemented with glucose at 37°C is depicted.

Supplementary Figure S2 Proportion of fatty acid classes identified in *T. fusca* cultures. Mole percent of total fatty acids produced by *T. fusca* in different growth conditions, organized by lipid species. “*n-*FA” indicates linear fatty acid chain.

Supplementary Figure S3 Proportion of fatty acid chain lengths identified in *T. fusca* cultures. Mole percent of total fatty acids produced by *T. fusca* in different growth conditions, organized by chain length.
