# Supplementary figures and images for: The Effects of Carbon Source and Growth Temperature on the Fatty Acid Profiles of Thermobifida fusca
Source: Front Mol Biosci. 2022 Jun 1;9:896226. doi: 10.3389/fmolb.2022.896226 (PMC9198275; doi:10.3389/fmolb.2022.896226)

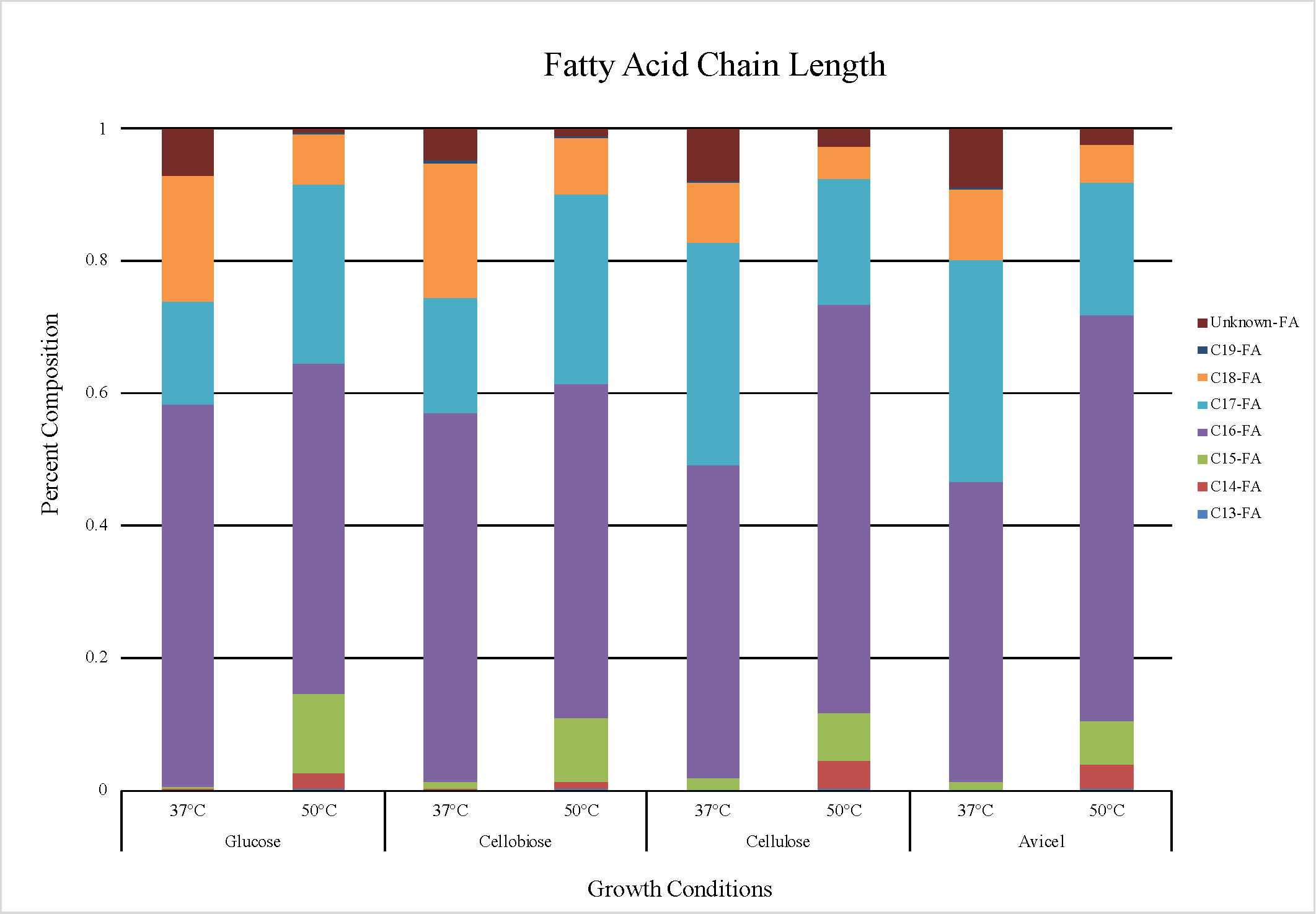

Supplement: Supplementary file 1 [file Image3.TIFF]

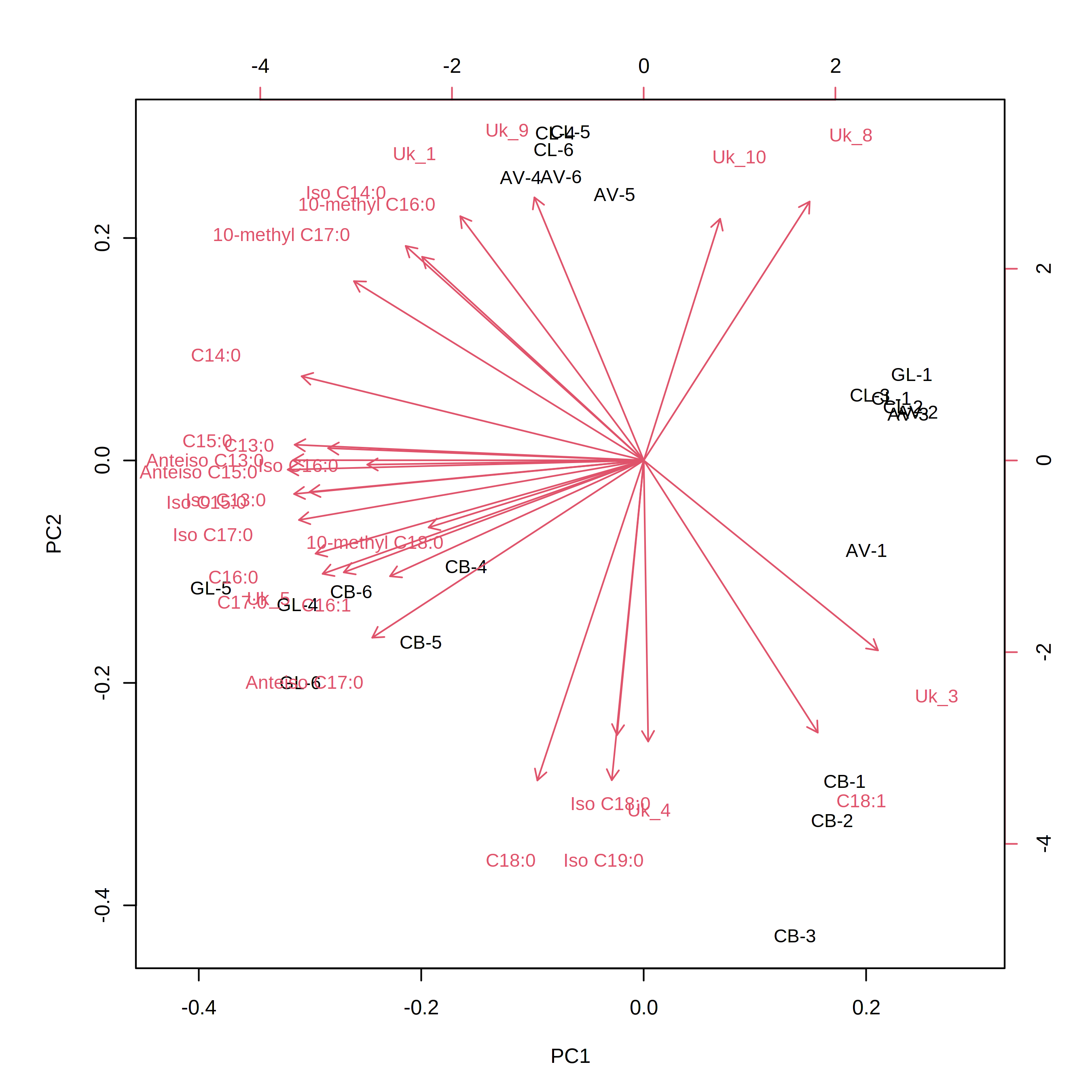

Supplement: Supplementary file 2 [file Image1.TIFF]

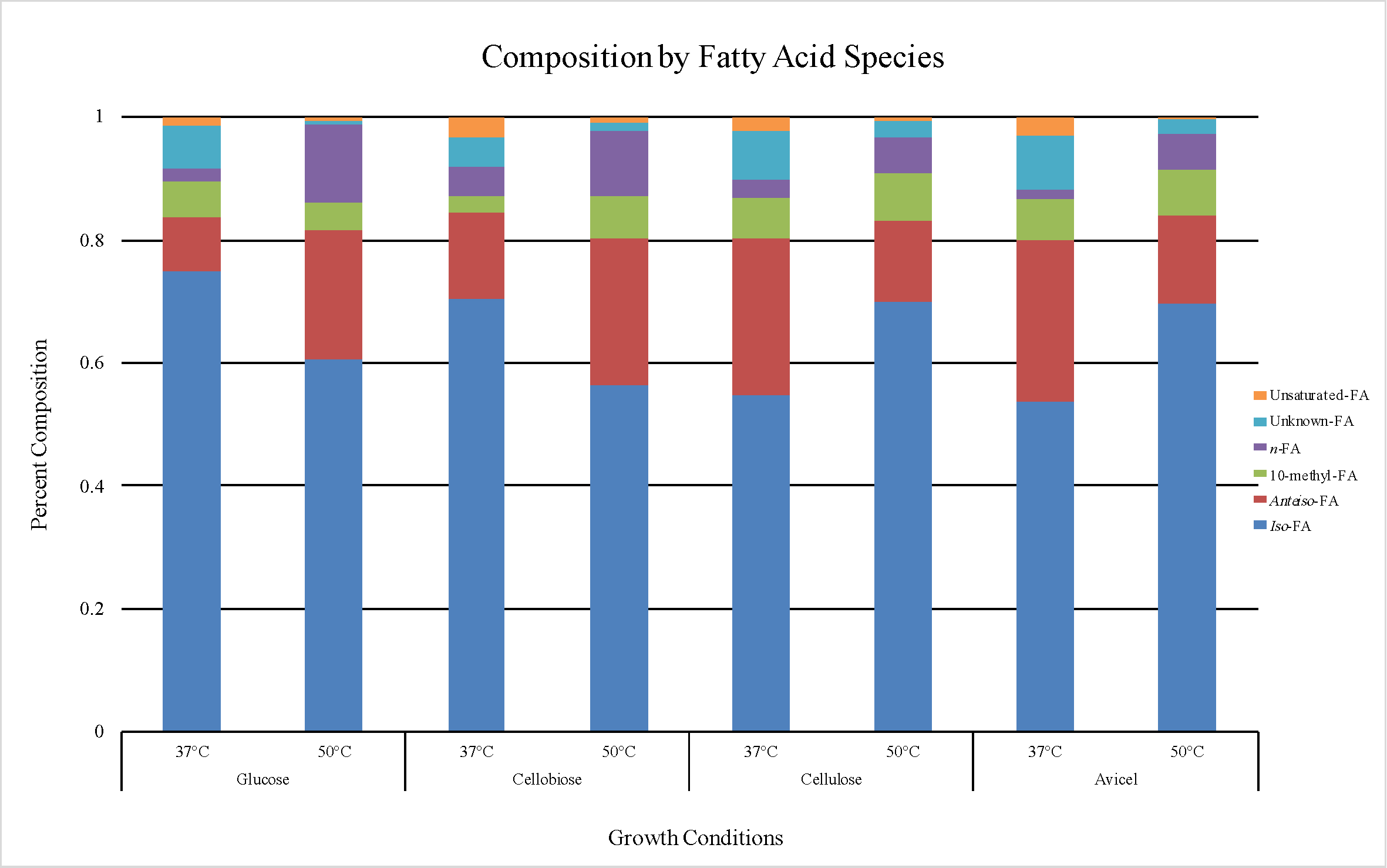

Supplement: Supplementary file 3 [file Image2.TIFF]
